# Interaction of Physical Exposures and Occupational Factors on Sickness Absence in Automotive Industry Workers

**DOI:** 10.5539/gjhs.v7n6p276

**Published:** 2015-04-23

**Authors:** Fateme Valirad, Mostafa Ghaffari, Alireza Abdi, Mirsaeed Attarchi, Seyed Farzin Mircheraghi, Saber Mohammadi

**Affiliations:** 1 Occupational Medicine Department, Faculty of Medicine- Iran University of Medical Sciences, Tehran, Iran; 2 Brain and Spinal Injury Research Center (BASIR), Imam Khomeini Hospital, Tehran University of Medical Sciences, Tehran, Iran; 3 Internal Medicine Departments, Faculty of Medicine, Gonabad University of Medical Sciences, Gonabad, Iran

**Keywords:** sickness absence, physical exposure, risk factors, shift work, blue collar

## Abstract

**Introduction:**

Increased sickness absence in recent years has been a trouble making issue in industrial society. Identify the causes of sickness absence and its influencing factors, is an important step to control and reduce its associated complications and costs. The aim of this study was to evaluate main factors associated with the incidence of sickness absence.

**Procedure:**

In 2012, a cross-sectional study on 758 employees of a car accessories producing company was applied and relevant information about the number of days and episodes of sickness absence, Disease resulting in absence from work, personal features, occupational factors and physical exposures were collected. To determine risk factors associated with sickness absence, Logistic regression analysis was used.

**Results:**

The most common diseases leading to sickness absence in order of frequency were Respiratory diseases, musculoskeletal disorders, gastrointestinal diseases and injuries at work. Musculoskeletal disorders increased the danger of long term absence by 4/33 times. Blue collar and shift works were the most important occupational factors associated with the incidence of sickness absence. The main physical factors that affect incidence of sickness absence were frequent bending-twisting and heavy lifting.

**Conclusion:**

Identifying controllable factors of sickness absence and trying to prevent and modify them such as compliance of ergonomic principals to decrease physical can be effective in reducing sickness absence.

## 1. Introduction

Due to negative impacts on labor productivity, cumbersome costs and interference with production quality (d’Errico et al., 2012; Kremer et al., 2010; Ferrie et al., 2009; Head et al., 2008; Kivimaki et al., 2007; Zenz, 1994), sickness absence from work has attracted more attention in recent years and has become an important issue in industrialized societies (Lund et al., 2009; Zenz, 1994). Sickness Absence is a major socio-economic problem and imposes high costs on societies (d’Errico et al., 2012; Kremer et al., 2010; Ferrie et al., 2009; Head et al., 2008; Kivimaki et al., 2007; Rahme et al., 2006; Zenz, 1994). For example, during 2013 in UK, 131 million working days lost due to sickness absence (Salus, 2011) and in 2012, 227 billion dollars were disbursed in United States of America, only to reduce productivity losses due to sickness absence (Forbes, 2012).

Sickness Absence is a complex situation and has a multi-factorial etiology (Ferreira et al., 2012; Virtanen et al., 2008). According to previous studies, factors such as individual characteristics and occupational factors, affect the incidence and severity of Sickness Absence (d’Errico et al., 2012; Kristensen et al., 2010; Lund et al., 2009; Niedhammer et al., 2008; Virtanen et al., 2008; Duijts et al., 2007; Dekkers-Sanchez et al., 2007; Ose, 2005; Labriola et al., 2006; Bultmann et al., 2005; Vingrd et al., 2005; Zenz, 1994). Obviously, many of these factors are controllable and preventable. That’s why many studies have been carried out to identify factors associated with the sickness absence in different industries and occupations. In a study conducted by Alavinia et al. (2009) authors found that obesity, smoking and manual materials handling were important risk factors for moderate and long durations of sickness absences.

Given the particular importance of identifying the causes of absence from work and factors affecting it, further studies in this area would be beneficial. The aim of our study was to identify the causes of absence from work and examines the impact of individual factors, occupational and physical exposures on the incidence of Sickness Absence in the automotive industry workers.

## 2. Methods

### 2.1 Study Design and Population

This Cross-sectional study has been carried out since November 2011 till October 2012 on the employees of a car accessories production company in Tehran, Iran. Workers who had at least one year of experience were recruited on a voluntary basis. Distribution, collection and monitoring of response to the questionnaires performed by a general practitioner working in the HSE Unit. Some parts of job information such as dates of employment, working location and shift work status were extracted from company`s staffing and recruiting unit. This study was approved by the ethics committee of Iran University of medical sciences.

### 2.2 Sickness Absence

The SA data were extracted in 2011 from computerized system of HSE unit. This data include number of days missed, number of episodes of absence and illness resulting in sickness absence. The absences duration were divided into two groups of short term (<3 day) and long term (≥3day) (Virtanen et al., 2008). Diseases were classified according to International Classification of Diseases version ten (ICD10) (WHO, 2010).

### 2.3 Questionnaire and Determinants

A self-administered questionnaire was used to collect information regarding the following variables: 1- Socio-demographic factors such as age, sex, marital status and education level. 2- Lifestyle including smoking (number of years and number of cigarettes per day), height and weight were used to calculate BMI and subjects based on BMI number are divided into three groups of normal: BMI<25, overweight: 25 ≤BMI <30 and obese: BMI≥ 30 (Ferreira et al., 2012). 3- Physical exposure: in order to assess this variable, the MUSIC (musculoskeletal intervention center)-Norrtalje questionnaire (Alipour et al., 2007) which contains 12 questions was used. The first question was a Visual Analog Scale (VAS) that evaluated the hard work of the workers. other questions assessed the duration of sitting at work, working in front of a computer screen and working with vibrating tools, vibration exposure levels, frequency of bending and twisting of the back, Heavy lifting and so on. The answers to these questions should be chosen on a 5-point likert scale. Answer as never, a quarter of times and total score less than or equal to 30 were considered as low physical exposure and Answers as half, three quarters, always and total score greater than 30 were considered as high physical exposure.

### 2.4 Statistical Analysis

Sickness absence was our dependent variable in this study and independent variables were individual, socio-demographic, occupational and physical exposures. No absences subjects were considered as the reference group. Chi- square test was applied to compare qualitative variables and Binary logistic regression analysis was employed to determine the variables associated with SA. In all tests, the significant level was considered as 0.05 with confidence interval at 95 % and data statistical analysis was carried out with SPSS software version 13.

## 3. Results

In this study, 956 questionnaires distributed among employees and 790 questionnaires were returned. The response rate was 82.63%. 31 questionnaires were excluded due to incomplete data and one questionnaire due to absence related to pregnancy. Finally, 758 questionnaires were analyzed. The average age of the sample was 35.08 (±7.03 SD) years and the range was from 21 years to 63 years. 94.3% of subjects were male. 51.7% of cases had abnormal BMI (overweight and obesity). 72% of participants were shift workers and 74% were blue collars.

The analysis of sickness absence showed that the 49.86% (n=378) of respondents had sickness absence and about 60.84% (n=230) of this group had one absence episode, 30.15% (n=114) had two absence episodes and 9% (n=34) had three absence episodes. Mean duration of short term sickness absences was 1.69 days (Standard Deviation: 0.73) and Mean duration of long term sickness absences was 10.92 days (Standard Deviation: 13.7). The most common diseases leading to absence from work in order of frequency were upper respiratory diseases (77.08%), musculoskeletal disorders (8.88%), gastrointestinal disease (5.15%) and injuries (4.3%). The remaining other diseases percentages were low. Respiratory diseases and gastrointestinal disorders resulting in short term sickness absences while the musculoskeletal disorders and injuries leading to long term sickness absence periods. In this study, the results showed that the musculoskeletal diseases increased the risk of long term sickness absence 4.33 times [(95%CI=2.44-7.68), p<0.001] and this risk for injuries was 1.95 times [(95%CI=1.43-2.64), p<0.001]. In Tables 1 and 2, descriptive results are shown in short and long terms sickness absence groups.

**Table 1:** Descriptive statistics of individual, lifestyle and occupational characteristic

Variable	No SA*		Short term SA		Long term SA	

	Mean (SD)	N (%)	Mean (SD)	N (%)	Mean (SD)	N (%)
**SA day**	0		1.69 (*±*0.73)	…	10.92(*±*13.7)	
**Sex**	…					
Female		29 (7.6%)	…	12(3.5%)	…	2(5.1%)
Male		351(92.4%)		327(96.5%)		37(94.9%)
**Age**				…		
20-30	27.87(±1.93)	113(29.7%)	27.68(±1.88)	107 (31.6%)	28.0(±2.00)	7 (40%)
>30	35.31(±2.89)	267(70.3)	35.24(±2.91)	232(68.4%)	35.21(±2.80)	22 (60%)
**Marital status**						
Married	…	333(87.6%)	…	299 (88.2%)	…	35(89.7%)
Single		47 (12.4%)		40 (11.8%)		4(10.3%)
**Education**						
University	…	105(27.6%)	…	281 (83.1%)	…	30(77.5%)
No university		275(72.4%)		57(16.9%)		9(22.5%)
**BMI**						
Normal	23.09(±1.50)	175(46.1%)	23.06(±1.63)	169 (49.9%)	23.18(±1.50)	22(56.4%)
Overweight	27.22(±1.32)	168(44.2%)	26.81(±1.36)	149 (44%)	26.95(±1.36)	11(28.2%)
Obesity	32.33(±2.03)	37 (9.7%)	32.70(±2.21)	21(6.2%)	31.48(±1.18)	6(15.4%)
**Smoking**						
Never		229(78.7%)		266 (78.5%)		31(79.5%)
Current	1.05 (±4.62)	81 (21.3%)	0.66 (±2.37)	73 (21.5%)	0.32 (±6.36)	8(20.5%)
Pack/year						
**Job tenure**						
<7year	3.73(±2.13)	208(54.7%)	4.07(±2.24)	180(53.3%)	4.35(±2.10)	23(57.5%)
≥7years	11.77(±3.31)	172(45.3%)	11.87(±3.24)	153(46.7%)	9.94(±2.90)	17(42.5%)
**Work type**						
White collar	…	137(36.1%)		55(16.2%)	…	6(15.4%)
Blue collar		243(63.9%)		284 (83.8%)		33(84.6%)
**Work schedule**						
Day (fixed)	…	143(37.6%)		61(18%)	…	7(17.9%)
Shift work		237(62.4%)		278 (82%)		32(84.6%)

* SA: sickness absence.

**Table 2:** Frequency distribution of physical exposures

Variable	No SA*	Short term SA	Long term SA

	N (%)	N (%)	N (%)
**Physical hard work**			
Low	77 (20.3%)	41 (12.1%)	3 (7.7%)
High	303 (79.7%)	297 (87.9%)	36 (92.3%)
**Sustained sitting work**			
Low	254 (66.8%)	240 (71.0%)	28 (71.8%)
High	126 (33.2%)	98 (29.0%)	11 (28.2%)
**Work in front of LCD!**			
Low	294 (77.4%)	291 (86.1%)	34 (87.2%)
High	86 (22.6%)	47 (13.9%)	5 (12.8%)
**Vibrating surface**			
Low	328 (86.3%)	283 (83.7%)	30 (76.9%)
High	52 (13.7%)	55 (16.3%)	9 (23.1%)
**Vibrating tools**			
Low	302 (79.5%)	261 (77.2%)	33 (84.6%)
High	78 (20.5%)	77 (22.8%)	6 (15.4%)
**Hand below the knee**			
Low	295 (77.6%)	263 (77.8%)	25 (64.1%)
High	105 (27.6%)	75 (22.2%)	14 (35.9%)
**Bending - twisting**			
Low	175 (46.1%)	131 (38.8%)	12 (30.8%)
High	205 (53.9%)	207 (61.2%)	26 (66.7%)
**Heavy Lifting**			
Low	168 (44.2%)	121 (35.8%)	13 (40.0%)
High	212 (55.8%)	217 (64.2%)	26 (65.0%)
**Hand above shoulder**			
Low	275 (72.4%)	228 (67.5%)	22 (56.4%)
High	105 (27.6%)	110 (32.5%)	17 (43.6%)
**Repetitive movement**			
Low	120 (31.6%)	97 (28.7%)	12 (30.8%)
High	260 (68.4%)	241 (71.3%)	27 (69.2%)
**Sensitive work**			
Low	225 (59.2%)	180 (53.3%)	20 (51.3%)
High	155 (40.8%)	158 (46.7%)	19 (48.7%)
**Total physical score**			
Low	212 (55.8%)	161 (47.6%)	17 (43.6%)
High	168 (44.2%)	177 (52.4%)	22 (56.4%)

* SA: sickness absence.

Chi-square analysis and multivariate analysis (Binary Logistic Regression) results are shown in tables III. According to this table: male workers had more sickness absence than female workers and this gender differences was more evident in the short-term absence [OR=2.25(95% CI=1.13-4.48), p=0.018].Workers with non-university education had 1.8 times more short-term absences. In our study, the highest OR in incidence of sickness absence was associated with two occupational factors: blue collar [OR=3.38 (95%CI=1.39-8.24), p=0.005] and shift works [OR=2.78 (95%CI=1.2-6.48), p=0.013].These variables were the most important risk factors in incidence of short and long term sickness absences. Among the physical exposure variables, risk factors associated with the higher incidence of sickness absence were physical hard work [OR=3.05 (95%CI=1.01-10.1), p=0.049], working in front of computer screen [OR=1.82(95%CI=1.23-2.69), p=0.002], over shoulder working [OR=1.99(95%CI=1.02-3.81), p=0.04], frequent bending -twisting [OR=1.86 (95%CI=1.091-4.02), p=0.008], and heavy lifting [OR=1.64 (95%CI=1.19-2.25), p=0.002].

**Table 3:** Chi-square and logistic regression results for risk factors of sickness absence.

Variable	Short term SA*		Long term SA			

	**OR(95% CI) P**	P	**OR(95% CI)**	P	LR**	P
**Sex**						
Female (ref)	1		1		1	
Male	2.25(1.13-4.48)	0.018	1.52(0.35-6.66)	0.75	1.07(0.50-2.25)	0.85
**Age**						
20-30	1		1			
>30	2.77(1.002-7.77)	0.59	1.92(0.96-4.90)	0.68		
**Marital status**			
Married(ref)	1		1			
Single	1.81(0.61-5.35)	0.27	1.35(0.46-3.96)	0.58		
**Education level**						
University (ref)	1		1		1		
No university	1.89 (1.31-2.72)	<0.001	1.21 (0.33-1.28)	0.60	1.09 (0.64-1.53)	0.77
**BMI**						
<25(ref)	1		1			
≥25	0.86 ( 0.64 – 1.16)	0.34	0.66 (0.57-2.56)	0.21		
**Smoking**						
Never (ref)	1		1			
Current	1.04 (0.73-1.49)	0.80	1.34 (0.57-3.14)	0.49		
**Work type**			
White collar(ref)	1		1		1		
Blue collar	2.98 (2.08-4.27)	<0.001	3.38 (1.39-8.24)	0.005	2.03 (1.30-3.14)	0.002*
**Work schedule**						
Day work(ref)	1		1		1		
Shift work	2.76 (1.96-3.93)	<0.001	2.78 (1.2-6.48)	0.013	1.74 (1.13-2.65)	0.009*
**Physical hard work**						
Low(ref)	1		1		1		
High	1.84 (1.22-2.78)	0.003	3.05 (1.01-10.1)	0.05	1.23 (0.78-1.94)	0.35
**Sitting work**						
Low (ref)	1		1			
High	1.22 (0.89-1.68)	0.21	1.24 (0.61-2.50)	0.54		
**Work in front of LCD**			
Low (ref)	1		1		1		
High	1.82 (1.23-2.69)	0.002	1.74 (0.71-4.28)	0.21	1.39 (0.8-2.42)	0.24
**Vibrating surface**						
Low (ref)	1		1			
High	1.19 (0.79-1.79)	0.39	1.81 (0.81-4.02)	0.14		
**Vibrating tools**						
Low(ref)	1		1			
High	1.13 (0.79-1.62)	0.47	1.30 (0.55-3.04)	0.53		
**Hand below the knee**			
Low(ref)	1		1			
High	1.00 (0.70-1.42)	0.98	1.94 (0.96-3.90)	0.58		
**Bending- twisting**						
Low(ref)	1		1		1		
High	1.37 (1.01-1.84)	0.038	1.86 (1.09-4.02)	0.008	1.85 (1.56-3.29)	0.046*
**Heavy Lifting**						
Low(ref)	1		1		1		
High	1.64 (1.19-2.25)	0.002	1.47 (0.74-2.90)	0.26	1.06 (1.09-1.63)	0.027*
**Over shoulder**						
Low(ref)	1		1			
High	1.25 (0.91-1.73)	0.15	1.99 (1.02-3.81)	0.04		
**Repetitive movement**						
Low(ref)	1		1			
High	1.16 (0.84-1.60)	0.34	1.05 (0.51-2.14)	0.89		
**Sensitive work**						
Low(ref)	1		1			
High	1.22 (0.96-1.64)	0.17	1.32 (0.68-2.55)	0.40		
**Total physical score**						
Low(ref)	1		1		1		
High	1.35 (1.08-1.81)	0.044	1.58 (0.84-3.07)	0.17	1.06 (0.77-1.46)	0.71

* SA: sickness absence.

** LR: logistic regression.

In regression analysis in the final multivariate model, blue collar [OR=2.03 (95%CI=1.30-3.14), p=0.002], shift work [OR=1.74 (95%CI=1.13-2.65), p=0.009], Bending-twisting [OR=1.85 (95%CI=1.56-3.29), p=0.009], and heavy lifting [OR=1.09 (95%CI=1.09-1.63), p=0.027] remained significant after adjusted for Confounding factors.

## 4. Discussion

In this study, we assessed the pattern of sickness absence in automotive industry workers and evaluated the impact of individual factors, occupational and physical exposures on this outcome. Our study showed that Short term sickness absences were associated with minor diseases while the long-term sickness absences were due to serious disorders which caused inability to work.

The most common causes of absence from work in order of frequency were respiratory illnesses (such as common colds and Influenza), musculoskeletal diseases, digestive diseases (e.g. gastroenteritis) and injuries. Musculoskeletal disorders increased the danger of long term sickness absence by 4/33 times and injuries by 1.95 times. These results are consistent with the results of some previous studies. In one study, the main reason for the sickness absence were respiratory diseases, musculoskeletal disorders, gastrointestinal diseases and injuries (Rahme et al., 2006) and in another study conducted by, the most common causes of sickness absence were colds and influenza and the highest proportion of days lost was related to musculoskeletal disorders (Kremer et al., 2010). (Lund et al., 2009) and as well as (Roberto Ferreira et al., 2012) obtained similar results. Colds and flu are prevalent respiratory diseases. The viral nature of this disease increases the risk of contagion to others. Employees usually work in closed spaces with poor ventilation that leads to an increased episode of disease transmission. Auto industry imposes high physical exposure and poor ergonomic state on workers. Long-term exposure to these conditions can superpose workers at risk for musculoskeletal problems. Injuries results in most working-day lost. Fractures (a common group of injury in our study) require a long time to heal, and during this time, worker can’t work and it results in long term absence from work.Our findings showed that gender, education level and aging were significantly effective on occurrence of absence from work. These findings are compatible with the results of previous studies. For instance, one research found that the risk of sickness absence in male workers was higher than females (Kivimaki et al., 2002). Similar results in a study conducted in Sweden confirmed this gender differences (Lund et al., 2009). In a cross-sectional study demonstrated that sickness absence declines with increasing level of education (Aaviksoo et al., 2013). In a study, association between increased sickness absence and aging was found (Alavinia et al., 2009).

Among the occupational factors, blue collar and shift works were the most important risk factors for incidence of absenteeism. White collar employees were not in contact with harmful hazards in the manufacturing sector and therefore had lower sickness absence. Shift working disturbances of the circadian rhythm system and thus reduced wellbeing, increase morbidity and the risk of many diseases (Rom, 2007). Some previous studies showed that blue collar workers had more sickness absence than white collar workers (Aaviksoo et al., 2013; Alavinia et al., 2009). The results of our study show that physical exposures like heavy lifting, bending-twisting of lumbar and hard physical work are associated with occurrence of sickness absence. This correlation may be due to increase incidence of musculoskeletal disorders. In previous studies the relationship between physical factors and sickness absence was seen: exposure to ergonomic factors such as heavy lifting repetitive movements can leads to increased sickness absence (d’Errico et al., 2012). Severe back bending, over shoulder working and heavy lifting were main factors causing sickness absence (Lond et al., 2009). In one study, physical exposures, lifting and intense physical conditions affect short term and long term sickness absences (Alavinia et al., 2009). There was significant relationship between physical exposures and sickness absence (Labriola et al., 2006; Aaviksoo et al., 2013).

In our study, data related to sickness absence was obtained from a computerized record system; thus were reliable and had no recall bias. But because of the cross-sectional nature of the study, identifying the causal relationship between risk factors and sickness absence was not possible. The healthy worker effect may alter the pattern of diseases due to the early departure of workers suffering from debilitating illnesses.

Finally, in this study we found that physical exposures (frequent bending-twisting and heavy lifting) accompanied by other occupational exposures (shift working and work as a blue collar worker) and also some individual factors are the most prominent factors affecting the incidence of sickness absence. Thus special attention should be paid to control these risk factors. Weight loss motivating policies, encouraging smoking cessation, educating workers, improving ergonomic principals and working conditions would be beneficial.
